# A Schiff Base Fluorescence Enhancement Probe for Fe(III) and Its Sensing Applications in Cancer Cells

**DOI:** 10.3390/s19112500

**Published:** 2019-05-31

**Authors:** Na Hee Kim, Junho Lee, Sungnam Park, Junyang Jung, Dokyoung Kim

**Affiliations:** 1 Department of Biomedical Science, Graduate School, Kyung Hee University, Seoul 02447, Korea; pionaheek@gmail.com; 2 Department of Chemistry, Korea University, Seoul 02841, Korea; pplkjh269@gmail.com (J.L.); spark8@korea.ac.kr (S.P.); 3 Department of Anatomy and Neurobiology, College of Medicine, Kyung Hee University, Seoul 02447, Korea; 4 Center for Converging Humanities, Korea University, Seoul 02841, Korea; 5 Medical Research Center for Bioreaction to Reactive Oxygen Species and Biomedical Science Institute, School of Medicine, Graduate School, Kyung Hee University, Seoul 02841, Korea

**Keywords:** fluorescent probe, Schiff base probe, iron ion sensing, D-A type fluorophore, bioimaging

## Abstract

We report a new Schiff base fluorescent probe which senses ferric ion, Fe(III), with a significant fluorescence enhancement response. The probe showed high sensitivity (0.8 ppb), and fast response time (<10 s) of Fe(III) in aqueous media. In addition, the probe showed the ability to sense Fe(III) in a HeLa cancer cell line, with very low cytotoxicity. As a new bio-imaging probe for Fe(III), it gave bright fluorescent images in confocal laser scanning microscopy (CLSM).

## 1. Introduction

Efficient detection of biologically important metal ions and the development of new fluorescent probes capable of this sensing is an important research topic in biology, chemical biology, environmental science, and medicine [1,2,3]. Iron (Fe) is an essential element among biologically important metal ions and plays crucial roles in blood production, heme synthesis, conversion of food to energy, immune cells proliferation and maturation, and normal cognitive function maintenance [4,5]. A high or low concentration of iron may induce disorders like Alzheimer’s disease (AD), Parkinson’s disease (PD), chronic kidney disease (CKD), and others [6,7]. Generally, iron exists in oxidation states between −2 and +7, but the most common are ferrous (Fe^2+^, Fe(II)) and ferric (Fe^3+^, Fe(III)) states [8]. In order to understand the active mechanism between the biological functions of ferrous and ferric, a detailed observation on each iron state is very important. For this reason, the development of a selective and sensitive molecular sensing system and fluorescent probe for each iron state has been highlighted.

To date, many fluorescent probes for Fe(III) have been reported (Figure S1, Supplementary Information) [9], and their approaches can be categorized into two; (i) selective binding between Fe(III) and ligand, and (ii) Fe(III)-induced chemical reactions. In the first approach, an imine-containing Schiff base fluorescent probe showed Fe(III)-sensing ability via monomer binding or excimer formation of blue-emitting dyes. However, low selectivity and interference from other heavy metal ions such as aluminum ions, Al(III), was considered disadvantageous (Figure S1). In some cases, the fluorescent emission at shorter wavelengths (λ_emi_ = 400–500 nm) or the turn-off property in Fe(III)-sensing, limits their usage in biological studies. Although such Schiff base fluorescence probes have drawbacks, they still have merits such as high sensitivity, fast response, reversibility, and have no side product generation [10]. The design of new Schiff moiety and fluorescent probes that fulfill Fe(III) sensing is very challenging due to strong fluorescence quenching effects of Fe(III) and difficult for the discrimination of Fe(II). In this study, we developed a new Schiff base fluorescent probe for Fe(III) sensing with fluorescence enhancement, and its bioimaging applications.

## 2. Materials and Methods

### 2.1. Materials

The chemical reagents were purchased from Aldrich (St. Louis, MO, USA), TCI (Tokyo, Japan), Alfa Aesar (Ward Hill, MA, USA), Deajung (Seoul, Korea), and Samchun (Seoul, Korea). Metal ions and amino acid (Aldrich, Alfa Aesar, Daejung, >97% purity): FeCl_3_, Fe(NO_3_)_3_, FeCl_2_, HgCl_2_, AgCl, CdCl_2_, NiCl_2_, CuCl_2_, PdCl_2_, ZnCl_2_, (C_2_H_5_)_3_PAuCl, KCl, CaCl_2_, NaCl, MgCl_2_, L-Cysteine, L-Glutamine, L-Lysine, Lysozyme. Hydrogen peroxide was purchased from TCI (35% in water, TCI, Tokyo, Japan). Commercially available reagents and anhydrous solvents were used without further purification. Chemical reactions were performed under argon atmosphere. Thin-layer chromatography (TLC) was performed using pre-coated silica gel 60F-254 glass plates (Merck KGaA, Darmstadt, Germany). 

### 2.2. UV/Vis Absorption and Emission Measurement

UV/Vis absorption spectra were obtained using spectrophotometer (Agilent Technologies Cary 8454, Santa Clara, CA, USA). Fluorescence spectrum were recorded on a spectro-fluorophotometer (SHIMADZU CORP. RF-6000, Kyoto, Japan) with a 1 cm standard quartz cell (internal volume of 1 mL, 108-000-10-40 (10 mm), 108-F-10-40 (10 × 4 mm); Hellma Analytics, Müllheim, Germany). The absorption and fluorescence spectrum were recorded at 10 μM concentration of **FeP-1** at 25 °C. 

### 2.3. NMR and Mass Analysis

^1^H NMR and ^13^C NMR spectra were measured with a Bruker AVANCE III 400 MHz (Bruker, Billerica, MA, USA). In the NMR spectra, the chemical shifts (δ) are reported in ppm, multiplicity are indicated by s (singlet), d (doublet), t (triplet), dd (double of doublets), and m (multiplet). Spectra were referenced to residual DMSO (2.50 ppm) in ^1^H NMR. High-resolution mass spectra were recorded on a JEOL JMS-700 spectrometer (JEOL, Tokyo, Japan) at the Korea Basic Science Center, Kyung-pook National University, and the values are reported in units of mass to charge (*m/z*).

### 2.4. Quantum Chemical Calculations

All calculations were carried out using the density functional theory (DFT) method at the APFD level with the 6-31+G(d,p) basis set as implemented in the Gaussian 16 package. The optimized structures and frontier orbitals (highest occupied molecular orbital; HOMO and lowest unoccupied molecular orbital; LUMO) of **FeP-1** and **FeP-1** + Fe(III) were obtained using the DFT method. The integral equation formalism polarizable continuum (IEF-PCM) model was used for solvation.

### 2.5. Cell Culture and CLSM Imaging of Cells

HeLa cell line was obtained from the Korean Cell Line Bank. Cells were cultured in Dulbecco’s modified Eagle’s media (Hyclone, Logan, UT, USA) supplemented with 10% fetal bovine serum (Hyclone) and 1% penicillin-streptomycin (Gibco). Cultures were incubated at 37 °C in humidified air containing 5% CO_2_. Approximately 5 × 10^4^ cells were seeded on 35 mm glass bottom confocal dishes (SPL Life Science, Gyeonggi-do, Korea) and incubated for 24 h. At the 80% confluency, the experiment was carried out. (i) **FeP-1**: **FeP-1** (50 μM) was treated to cells for 2 h. (ii) Fe(III): cells with the Fe(III) (50 μM) incubated for 2 h. (iii) **FeP-1** + Fe(III): cells pretreated the **FeP-1** (50 μM) for 2 h and then incubated with Fe (III) (50 μM) for 2 h at 37 °C in 5% CO_2_. Fluorescence images were visualized by a confocal laser scanning microscope (CLSM, LSM-800, Carl Zeiss, Germany). Excitation wavelength: 450 nm. Fluorescence detection channel; 455–600 nm. 

### 2.6. Cell Viability Assay

Approximately 1 × 10^5^ cells were seeded on a 96-well transparent bottom plate (SPL Life Science, Gyeonggi-do, Korea) and incubated for 24 h. After incubation, the cells were treated with DMSO as a control and **FeP-1** (0, 1, 3, 10, 30, 50 and 100 μM) for 24 h. The cell viability was analyzed by Vybrant® MTT Cell Proliferation Assay Kit (ThermoFisher, Waltham, MA, USA) following manufacturer’s instructions. The absorbance level was analyzed at 550 nm by microplate reader (Multiskan™ FC Microplate Photometer, ThermoFisher, Waltham, MA, USA). The culture medium was used as a control set.

## 3. Results and Discussion

**Figure 1:**
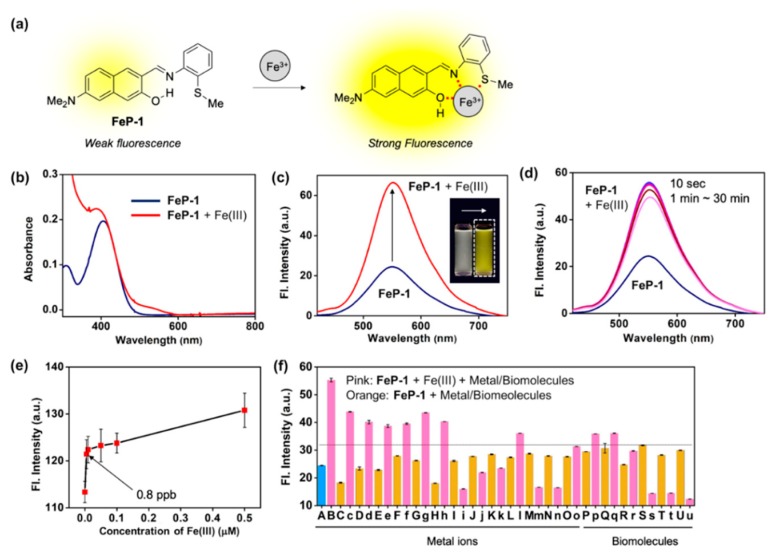
(**a**) Proposed sensing mechanism between Fe(III) and Schiff base fluorescence enhancement probe, **FeP-1**. (**b**,**c**) Absorption and emission spectra of **FeP-1** (10 μM) after addition of Fe(III) (20 eq). Inset photo: color change of the probe under UV light (365 nm) after addition of the Fe(III) (20 eq). Each spectrum was acquired in ethanol-water solution (EtOH:DI H_2_O = 4:6, *v/v*) after 1 min at 25 °C under excitation at 397 nm. (**d**) Time-dependent fluorescence spectra of **FeP-1** (10 μM) with Fe(III) (20 eq). Emission spectrum was measured at 10 s, 1, 3, 5, 10, 20, 30 min after mixing together. (**e**) A plot of fluorescence intensity (peak height at 550 nm) of **FeP-1** (0.5 μM) after addition of Fe(III) (0–0.5 μM). Mean and standard deviations was calculated in triplicate. (**f**) Fluorescence intensity plot (peak height at 550 nm) of **FeP-1** (10 μM) after addition of each metal ions (20 eq), amino acid (20 eq), and lysozyme (1–100 μg/mL) in ethanol-water solution (EtOH:DI H_2_O = 4:6, *v/v*), measured after 1 min at 25 °C. (A) **FeP-1**, (B) FeCl_3_, (C) FeCl_2_, (c) FeCl_2_ + FeCl_3_, (D) Hg(NO_3_)_2_, (d) Hg(NO_3_)_2_ + FeCl_3_, (E) AgCl, (e) AgCl + FeCl_3_, (F) CdCl_2_, (f) CdCl_2_ + FeCl_3_, (G) NiCl_2_, (g) NiCl_2_ + FeCl_3_, (H) CuCl_2_, (h) CuCl_2_ + FeCl_3_, (I) PdCl_2_, (i) PdCl_2_ + FeCl_3_, (J) ZnCl_2_, (j) ZnCl_2_ + FeCl_3_, (K) (C_2_H_5_)_3_PAuCl, (k) (C_2_H_5_)_3_PAuCl + FeCl_3_, (L) KCl, (l) KCl + FeCl_3_, (M) CaCl_2_, (m) CaCl_2_ + FeCl_3_, (N) NaCl, (n) NaCl + FeCl_3_, (O) MgCl_2_, (o) MgCl_2_ + FeCl_3_, (P) L-Cysteine, (p) L-Cysteine + FeCl_3_, (Q) L-Glutamine, (q) L-Glutamine + FeCl_3_, (R) L-Lysine, (r) L-Lysine + FeCl_3_, (S) Lysozyme 1 μg/mL, (s) Lysozyme 1 μg/mL + FeCl_3_, (T) Lysozyme 10 μg/mL, (t) Lysozyme 10 μg/mL + FeCl_3_, (U) Lysozyme 100 μg/mL, (u) Lysozyme 100 μg/mL + FeCl_3_. Means and standard deviations calculated from multiple measurements (n = 5). Dotted-line: Fe(III)-responsive signal threshold (at 32 a.u.). The emission spectrum was measured under excitation at 397 nm.

We recently focused on the development of a naphthalene-based donor (D)–acceptor (A) type fluorescent platform in order to monitor biologically important targets, such as enzyme activity, cell organelles, metal ions, and carcinogens [11,12,13,14]. During this research process, we found a new Schiff base fluorescent probe, **FeP-1**, that can sense Fe(III) with significant fluorescence enhancements (Figure 1a). This is the first report of a Schiff-type ligand on the D–A type fluorophore for Fe(III). We clearly identified the synthesis of the probe, sensing properties, and DFT calculations. To verify the application to biological studies, we demonstrated the tracking of endogenous as well as exogenous Fe(III) in HeLa cells via fluorescence imaging. Another key advantage was a low level of cytotoxicity.

The Schiff base probe, **FeP-1**, was prepared by one step synthesis, condensing 6-(dimethylamino)-3-hydroxy-2-naphth-aldehyde with 2-(methylthio)-aniline, and a catalytic amount of acetic acid in refluxing dichloromethane, which produced an ocher-colored solid at 69% yield (Scheme S1, Supplementary Information). The product was characterized by ^1^H/^13^C NMR and high-resolution mass spectrometry (Supplementary Data).

We discovered that the excited state intramolecular proton transfer (ESIPT) caused the fluorescence quenching of **FeP-1**, and the coordination with Fe(III) may suppress this pathway (Figure S2) [15,16,17]. Thus, the coordination of Fe(III) in the naphthol–imine–thiomethyl pocket of **FeP-1** would accompany both absorption and emission enhancements. This scenario was demonstrated through *in vitro* experiments as well as computational calculation, enabling us to sense Fe(III) as a form of fluorescence enhancement. 

A solution of **FeP-1** in aqueous media (DI H_2_O:EtOH = 6:4, *v/v*) exhibited weak fluorescence at an emission maximum of 550 nm. However, after being treated with Fe(III), it showed significant fluorescence enhancement (Figure 1b,c). In the different ethanol fractions (30–100%), the fluorescence intensity of **FeP-1** itself became higher as ethanol fraction increased, and the fluorescence enhancement factor toward Fe(III) also increased (>2 fold) (Figure S3). Time-course fluorescence changes for the mixture of **FeP-1** and Fe(III) was monitored in the given solution at 25 °C under excitation at the maximum absorption wavelength, 397 nm. As shown in Figure 1d, when Fe(III) was treated in a solution of **FeP-1**, a fluorescence enhancement was rapidly observed within 10 s, and it was saturated. A good linear relationship between the fluorescence intensity of **FeP-1** (10 μM) and Fe(III) was observed for a wide range of Fe(III) concentration (0–200 μM) (Figure S4), but an excessive amount of Fe(III) rather quenched the fluorescence. This data represent that the complex formation between **FeP-1** and Fe(III) is not corresponded to 1:1 binding. In the low concentration range of Fe(III), **FeP-1** displayed a high sensitivity; detection limit for 0.8 ppb (Figure 1e). Only Fe(III) enhanced the significant fluorescence of **FeP-1**, and other metal ions or enzymes showed a little responses (Figure 1f, Figure S5). A slight interference from some of metal ions and biomolecules was observed, but the signal enhancement mainly observed in the presence of Fe(III) (threshold at 32 a.u. in Figure 1f). A slightly decreased signal of **FeP-1** in the mixture of Fe(III) and metal ions (i.e., Pd(II), Zn(II), Au(III), Ca(II), Na(I), Mg(II)) was observed. Currently, we are focused on this phenomenon to understand the reason, and the information will be published somewhere. In addition, a decreased fluorescence signal of **FeP-1** with Fe(III) in the presence of lysozyme was also monitored, and the result might be caused from the aggregation factor of lysozyme in the given sensing media. 

Next, a selectivity of **FeP-1** toward Fe(III) not Fe(II) was double-checked by simply introducing oxidizing agent; Fe(II) to Fe(III). Significant fluorescence enhancement began to be observed after adding the oxidizing agent (H_2_O_2_ in this study) into the solution of **FeP-1** and Fe(II) (Figure S6). The optimal range for the fluorescence sensing of Fe(III) with **FeP-1** was found to be pH 4–8 including physiological pH (Figure S7). Additionally, no significant counter anion effect was confirmed by comparing the fluorescence signal response of **FeP-1** toward FeCl_3_ and Fe(NO_3_)_3_, respectively (Figure S8).

**Figure 2:**
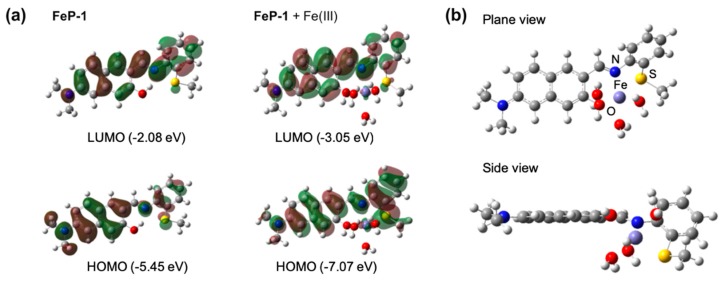
(**a**) The optimized molecular structures and HOMO/LUMO, and energy levels of **FeP-1**, **FeP-1** + Fe(III) complex by DFT calculations (APFD/6-31+G(d,p)). (**b**) Plane/side view of **FeP-1** + Fe(III) complex. Fe(III) complex contains three additional water molecules in DFT calculations.

The optimized molecular structures and HOMO-LUMO energy levels of **FeP-1** as well as **FeP-1** + Fe(III) complex were obtained by computational calculations (Figure 2a). In the **FeP-1** calculation, the intramolecular hydrogen bonding (H-bonding) between hydroxyl proton on the naphthalene backbone and imine was clearly observed in the Schiff ligand pocket, that can cause ESIPT-induced fluorescence quenching [16]. The localized electron distribution on the amine donor in HOMO, which is shifted to the imine moiety in LUMO, indicated the intramolecular charge transfer (ICT) character of D-A type fluorophores [18]. In contrast to **FeP-1**, **FeP-1** + Fe(III) complex showed that Fe(III) coordinates with Schiff base ligand; hydroxy, imine, and thiomethyl, with missing of H-bonding between naphthol and imine. In the side view of the **FeP-1** + Fe(III) complex, the thiomethyl-aniline showed a tilted conformation at an angle of 146°, from the naphthalene backbone to make a coordinate with Fe(III) (Figure 2b).

Before moving on to the bioimaging application, we verified the imine stability of **FeP-1** and its Fe(III) complex. Generally, an imine moiety could be hydrolyzed under acidic conditions or metal-coordination. To verify this factor, we conducted ^1^H NMR comparison for **FeP-1** and its incubation product with Fe(III). New peaks were not observed in this study, which indicates the stability of imine-moiety and fluorescence enhancement derived from the Fe(III) coordination, not hydrolysis (Figure S9). The imine stability of **FeP-1** was also tested in the sensing solution and cell lysate solution (whole cell lysate, HeLa cell, number of cells: 5 × 10^5^) in cell culture media (10% fetal bovine serum and 1% penicillin-streptomycin, w/o phenol red) at different temperatures (25 °C, 37 °C) during 60 min incubations. The stability of **FeP-1** was followed by change of absorption spectrum and intensity. In this test, we observed: (i) No changes in sensing solution, (ii) slightly increased intensity of **FeP-1** after 60 min incubation in the cell lysate solution without peak shift, which is correlated with intensity increment of **FeP-1** after binding of intracellular Fe(III). These data indicate sufficient stability of **FeP-1** in the sensing condition as well as cellular environment (Figure S10).

**Figure 3:**
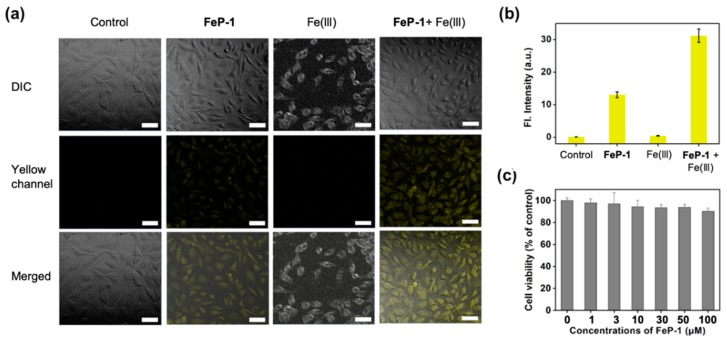
(**a**) CLSM images of the **FeP-1** (50 μM) in HeLa cells (magnification, ×20). Control: cells only; **FeP-1**: cells with the **FeP-1** (50 μM) incubated for 2 h; Fe(III): cells with the Fe(III) (50 μM) incubated for 2 h; **FeP-1** + Fe(III): cells pretreated the **FeP-1** (50 μM) for 2 h and then incubated with FeCl_3_ (50 μM) for 2 h. Excitation wavelengths 450 nm, and detection wavelengths channel were 455–600 nm, respectively. Scale bar is 20 μm. DIC: bright field, Yellow channel: fluorescence detection channel (455–600 nm), Merged: superimposed image of DIC and yellow channel. (**b**) The relative fluorescence intensity shown in panel (**a**). (**c**) Cell viability of **FeP-1**. Cells were incubated with 0–100 μM of **FeP-1** for 24 h in HeLa cells.

Next, **FeP-1** was applied to the bioimaging of Fe(III) in the cancer cell line, HeLa (immortalized human cervical cancer cell). When HeLa cells were incubated with **FeP-1**, a moderate fluorescence signal was observed in the cytosol within the yellow channel of confocal laser scanning microscopy (CLSM) (Figure 3a,b). When the exogenous source of Fe(III) (FeCl_3_, 50 μM) was post-incubated, the yellow emission was much brighter than that of the control group treated with **FeP-1**. The relative emission images in the experimental sets indicate that the selective sensing ability of **FeP-1** on Fe(III) in biological condition. The autofluorescence from the intrinsic fluorophores in the cells was not observed in the experiment condition (see control set in Figure 3a). In addition, **FeP-1** (0–100 μM) showed low cytotoxicity (<10%) in the cell viability assay, which holds great potential for further applications in bio-imaging as well as in biological mechanism studies (Figure 3c).

## 4. Conclusions

In summary, we have shown that a Schiff base fluorescent probe senses Fe(III). The probe shows a fluorescence enhancement at 550 nm upon addition of Fe(III), owing to a decrement of the ESIPT induced quenching effect. The introduction of the ESIPT approach into the Schiff base moiety of naphthalene-based D-A type fluorescent platform is new, and has a chance to develop into a more advanced system. Although the probe showed limitations such as sensing of Fe(III) in the mixture of organic solvents and interference from some metal ions, the probe showed high sensitivity with fast fluorescence response times with Fe(III). The application of probes for Fe(III) sensing in the cancer cell were successfully carried out. In this paper, we exclusively present a new Schiff ligand for Fe(III), which we believe it has the capability to serve as a practical sensor for Fe(III) detection in biomedical applications.

## Supplementary Materials

The following are available online at https://www.mdpi.com/1424-8220/19/11/2500/s1, Supplementary Figures: summary of known fluorescent probes, UV/Vis absorption and fluorescence spectra, ESIPT pathway, NMR analysis, HR-mass spectra.
