# Supplementary material for: A Schiff Base Fluorescence Enhancement Probe for Fe(III) and Its Sensing Applications in Cancer Cells
Source: Sensors (Basel). 2019 May 31;19(11):2500. doi: 10.3390/s19112500 (PMC6603573; doi:10.3390/s19112500)
Supplement: Supplementary file 1 [file sensors-19-02500-s001.pdf]

SUPPLEMENTARY INFORMATION

A Schiff Base Fluorescence Enhancement Probe for Fe(III) and Its Sensing Applications in Cancer Cells

Na Hee Kim, Junho Lee, Sungnam Park, Junyang Jung,* and Dokyoung Kim*

*Corresponding author. E-mail: dkim@khu.ac.kr (D. Kim), jjung@khu.ac.kr (J. Jung)

This file includes:

Synthesis of **FeP-1**

Supporting Scheme: Scheme S1

Supporting Figures: Fig. S1 to S10

^{1}H and ^{13}C NMR for **FeP-1**

High resolution mass spectra for **FeP-1**

References and notes

Synthesis of FeP-1.

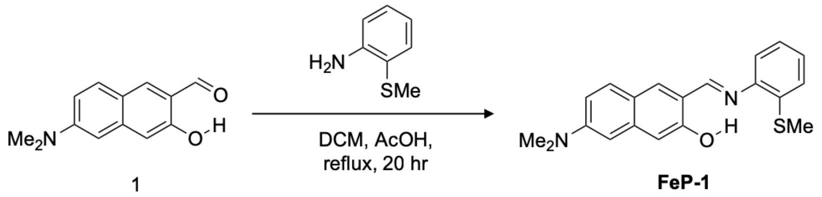

Scheme S1. Synthesis of **FeP-1**: Compound **1** (30 mg, 0.139 mmol), 2-(methylthio)-aniline (26.128 μL, 0.209 mmol, 1.5 eq), $MgSO_4$ (83.63 mg, 0.695 mmol, 5 eq), Dichloromethane (DCM, 2 mL), Acetic acid (0.1 mL), reflux for 20 hr, 69% yeld.

Synthesis of compound 1. We synthesized the compound 1 by following the reported method by our group. [Ref: 1. Kim, I.; Kim, D.; Sambasivan, S.; Ahn, K. H., Synthesis of π-Extended Coumarins and Evaluation of Their Precursors as Reactive Fluorescent Probes for Mercury Ions. *Asian J. Org. Chem*. **2012**, *1*, 60–64]

Synthesis of FeP-1. Compound **1** (30 mg, 0.139 mmol), 2-(methylthio)-aniline (26.128 μL, 0.209 mmol) and DCM (2 mL), magnesium sulfate (83.63 mg, 0.695 mmol), and acetic acid (0.1 mL) were combined at room temperature (25 °C). The reaction was held at reflux temperature overnight. The reaction mixture was washed with brine, and dried over $MgSO_4$. All the volatile components were removed in vacuo, and the crude residue was purified by column chromatography (*n*-hexane/ethyl acetate = 8:2) to give **FeP-1** (32.3 mg, 69%). ^{1}H NMR (400 MHz, DMSO-d$_6$): *δ* 12.55 (s, 1H), 8.97 (s, 1H), 7.73 (s, 1H), 7.70 (s, 1H), 7.44 (s, 1H), 7.42 (s, 1H), 7.31 (s, 1H), 7.30 (s, 1H), 7.07 (s, 1H), 7.05 (s, 1H), 6.98 (s, 1H), 3.05 (t, 6H), 2.47 (t, 3H) ; ^{13}C NMR (400 MHz, DMSO-d$_6$): *δ* 14.82, 40.44, 103.75, 109.00, 113.74, 116.96, 118.07, 120.89, 124.92, 125.25, 127.12, 129.83, 134.39, 135.06, 138.61, 145.70, 150.34, 157.40, 161.44. HRMS (*m*/*z*): $[M+Na]^+$ calcd for $C_{20}H_{20}N_2OS$, 336.1206; found, 336.1206.

Supporting Figures

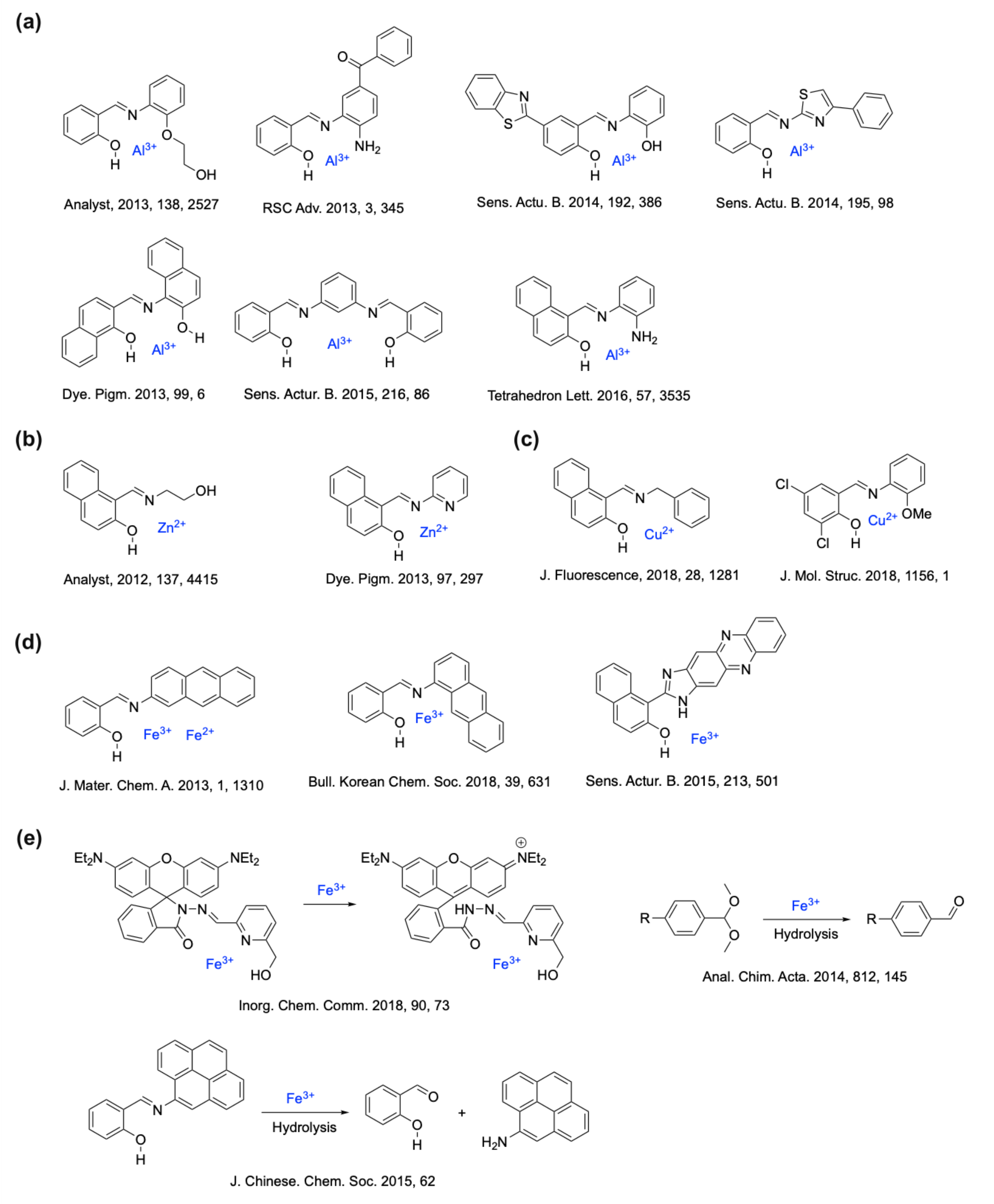

Figure S1. (a–d) Reported Schiff-based fluorescent probes, which have similar structure of **FeP-1**, for metal ions. (a) Al(III) probes. (b) Zn(II) probes. (c) Cu(II) Probes. (d) Fe(III), Fe(II) probes. (e) Reaction-based fluorescent probes for Fe(III). The references are fully addressed in References and notes.

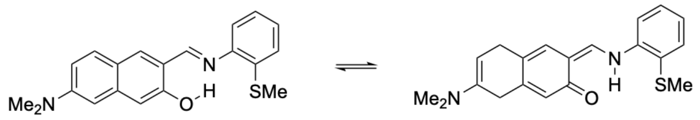

Figure S2. Excited state intramolecular proton transfer (ESIPT) mechanism of **FeP-1**.

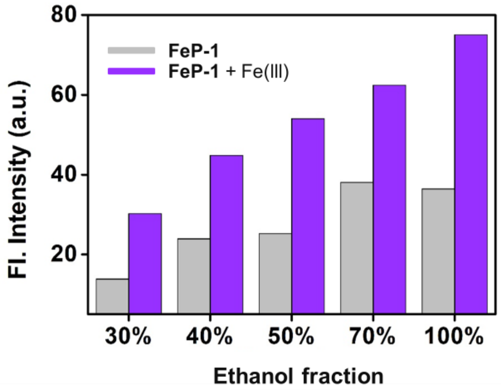

Figure S3. Fluorescence intensity plot (peak height at 550 nm) of **FeP-1** (10 μM) upon addition of Fe(III) (20 eq) in in various ethanol-water solution (30–100% EtOH), measured after mixing (within 1 min) at 25 °C. The emission spectrum was measured under excitation at 407 nm.

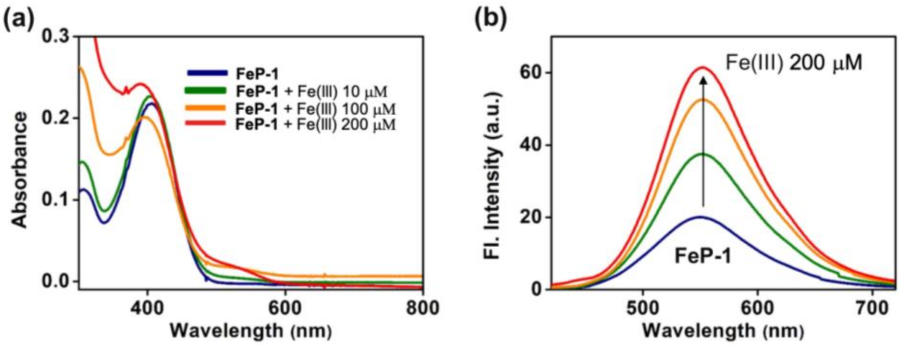

Figure S4. (**a**) Absorption and (**b**) Emission spectra of **FeP-1** (10 μM) upon addition of Fe(III) (0–200 μM) in ethanol-water solution (EtOH:DI H_2O = 4:6, *v*/*v*), measured after 1 min at 25 °C. The emission spectrum was measured under excitation at 397 nm.

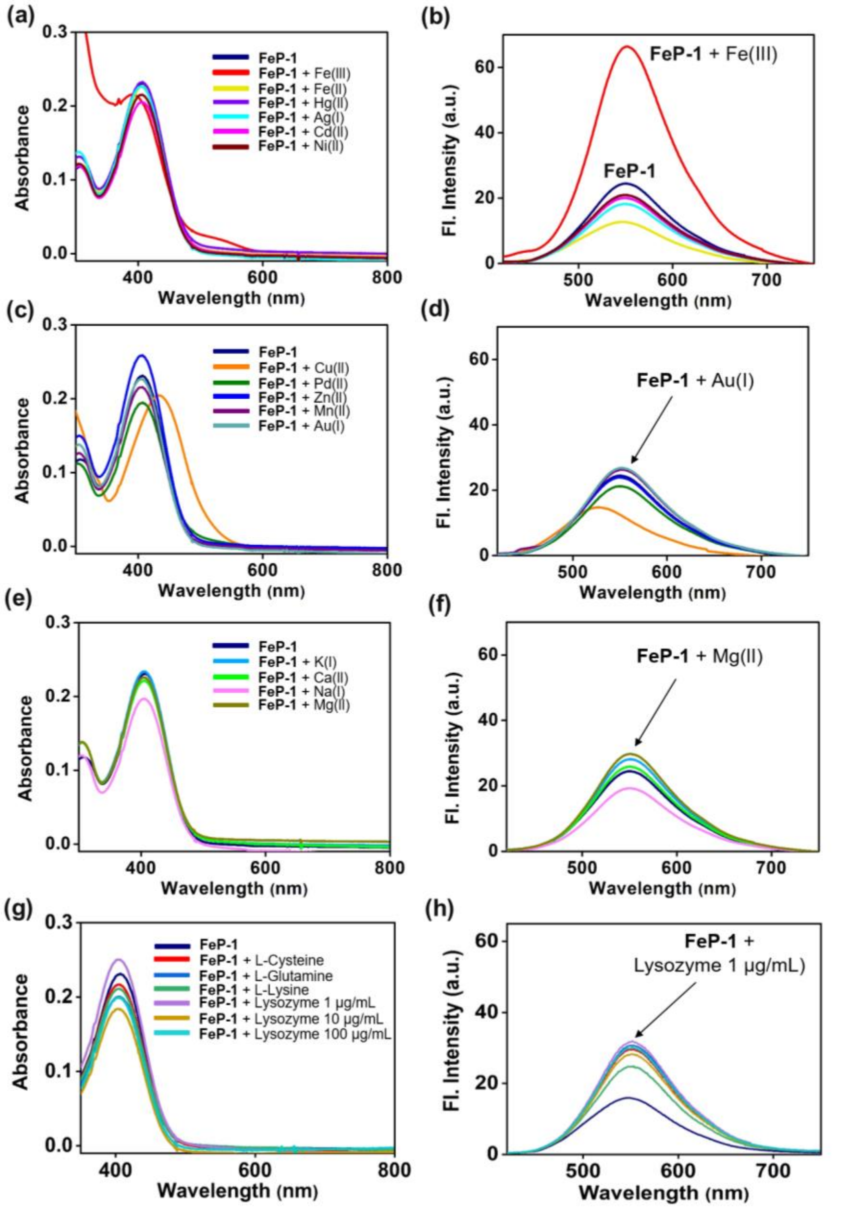

Figure S5. (a, c, e, g) Absorption and (b, d, f, h) Emission spectra of **FeP-1** (10 μM) upon addition of each metal ions (20 eq), amino acid (20 eq), and lysozyme (1–100 μg/mL) in ethanol-water solution (EtOH:DI H_2O = 4:6, *v*/*v*), measured after 1 min at 25 °C. Substrate: $FeCl_2$, $Hg(NO_3)_2$, AgCl, $CdCl_2$, $NiCl_2$, $CuCl_2$, $PdCl_2$, $ZnCl_2$, $MnCl_2$, KCl, $CaCl_2$, NaCl, $(C_2H_5)_3PAuCl$, $MgCl_2$, L-Cysteine, L-Glutamine, L-Lysine, Lysozyme 1μg/mL, Lysozyme 10 μg/mL, Lysozyme 100 μg/mL. The emission spectrum was measured under excitation at 397 nm.

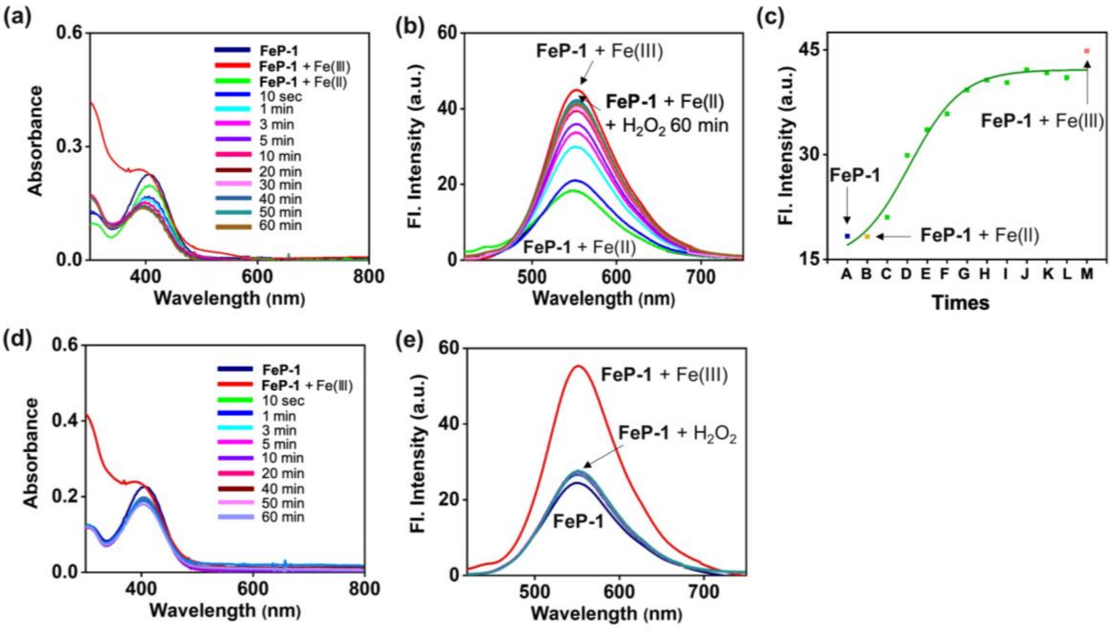

Figure S6. Time-dependent (a) absorption and (b) emission spectra changes of **FeP-1** (10 μM) with Fe(II) (20 eq) and H_2O_2 (20 eq) in ethanol-water solution (EtOH:DI H_2O = 4:6, *v/v*). Control: **FeP-1** (10 μM) with Fe(III). (c) Time-course emission intensity plot at maximum wavelength from panel (b). (A) **FeP-1**, (B) **FeP-1**+Fe(II), (C) **FeP-1**+Fe(ll)+Hydrogen peroxide; 10 s, (D) **FeP-1**+Fe(ll)+Hydrogen peroxide; 1 min, (E) **FeP-1**+Fe(ll)+Hydrogen peroxide; 3 min, (F) **FeP- 1**+Fe(ll)+Hydrogen peroxide; 5 min, (G) **FeP-1**+Fe(ll)+Hydrogen peroxide; 10 min, (H) **FeP-1**+Fe(ll)+Hydrogen peroxide; 20 min, (I) **FeP-1**+Fe(ll)+Hydrogen peroxide; 30 min, (J) **FeP-1**+Fe(ll)+Hydrogen peroxide; 40 min, (K) **FeP-1**+Fe(ll)+Hydrogen peroxide; 50 min, (L) **FeP-1**+Fe(ll)+Hydrogen peroxide; 60 min, (M) **FeP-1**+Fe(III). (d, e) Time-dependent absorption and emission spectra of **FeP-1** (10 μM) with H_2O_2 (20 eq). Absorption and emission spectrum was measured at 10 s, 1, 3, 5, 10, 20, 30 min after mixing together. Each spectrum was acquired in ethanol-water solution (EtOH:DI H_2O = 4:6, *v/v*) at 25 °C under excitation at 397 nm.

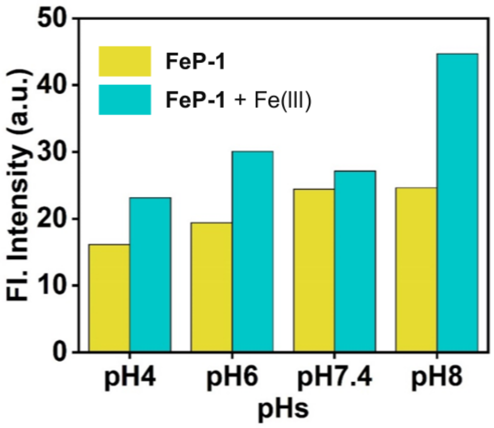

Figure S7. Emission intensity plot (peak height at 485 nm) of **FeP-1** (10 μM) upon addition of Fe(III) (20 eq) in various pHs (pH 4, 6, 7.4, 8), measured after 10 min at 25 °C. The emission spectra were measured under excitation at 397 nm.

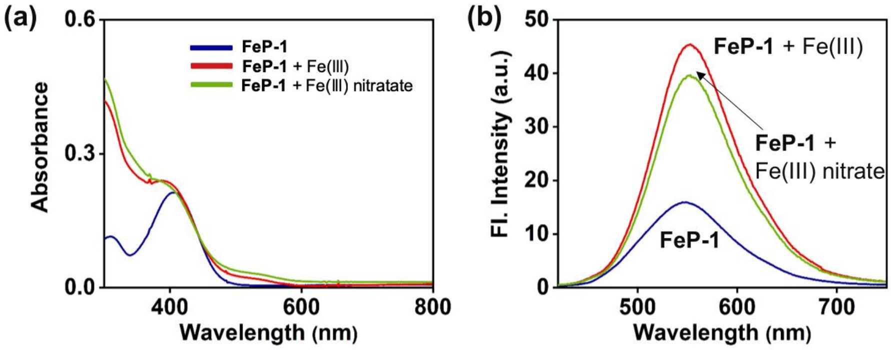

Figure S8. (a, b) Absorption and emission spectra of **FeP-1** (10 μM) upon addition of Fe(III) (20 eq), Fe(lll) nitrate (20 eq) in ethanol-water solution (EtOH:DI H_2O = 4:6, *v*/*v*) after 1 min at 25 °C.

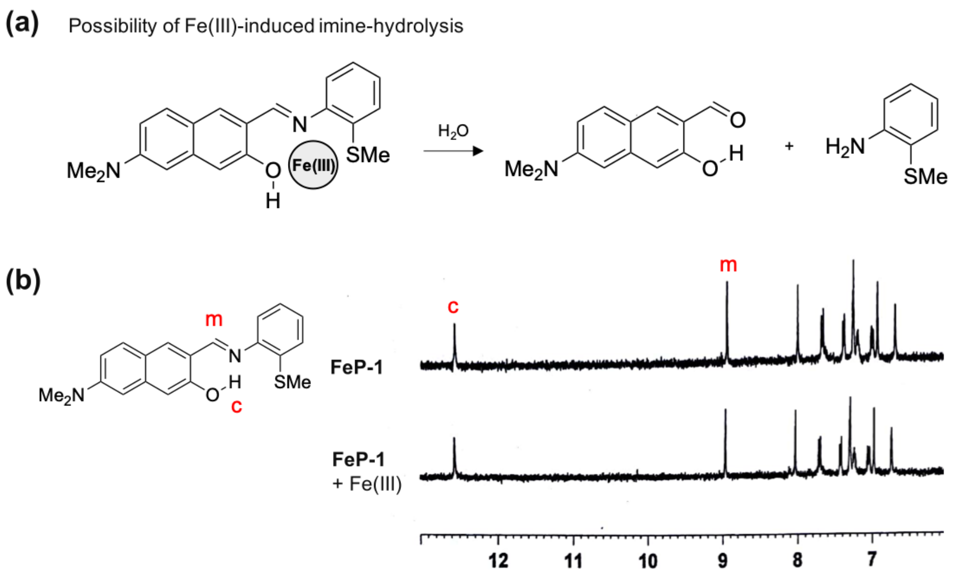

Figure S9. (a) Predicted hydrolysis pathway of **FeP-1**+Fe(III) in aqueous media. (b) ^{1}H NMR spectra for **FeP-1** (upper, 0.1 mg/mL) and its reaction product (bottom) with Fe(III) (1 mg/mL), 1 h incubation in ethanol-water solution (EtOH:DI H_2O = 4:6, *v*/*v*) at 25 °C, followed by extraction using dichloromethane. NMR solvent was DMSO-d_6.

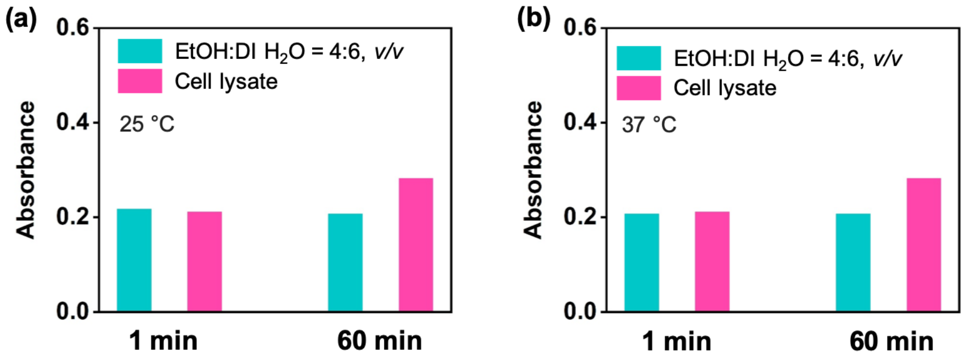

Figure S10. Absorption intensity of **FeP-1** (10 μM) in ethanol-water solution (EtOH:DI H_2O = 4:6, *v*/*v*) and HeLa cell lysate solution with incubation at (a) 25 °C and (b) 37 °C. The absorption intensity at 407 nm was plotted at 1 min and 60 min after adding the **FeP-1** to each solution. [Cell lysate solution] Lysis buffer (100 μL) was added to HeLa cells (5×10^5) in a 100 mm dish. After tapping the dish with hands, the resulting mixture was centrifuged at 14,000 rpm for 10 min, and the supernatant was collected. DMEM media (2 mL, 10% fetal bovine serum and 1% penicillin-streptomycin, w/o phenol red) was add to the supernatant.

^{1}H and ^{13}C NMR of FeP-1

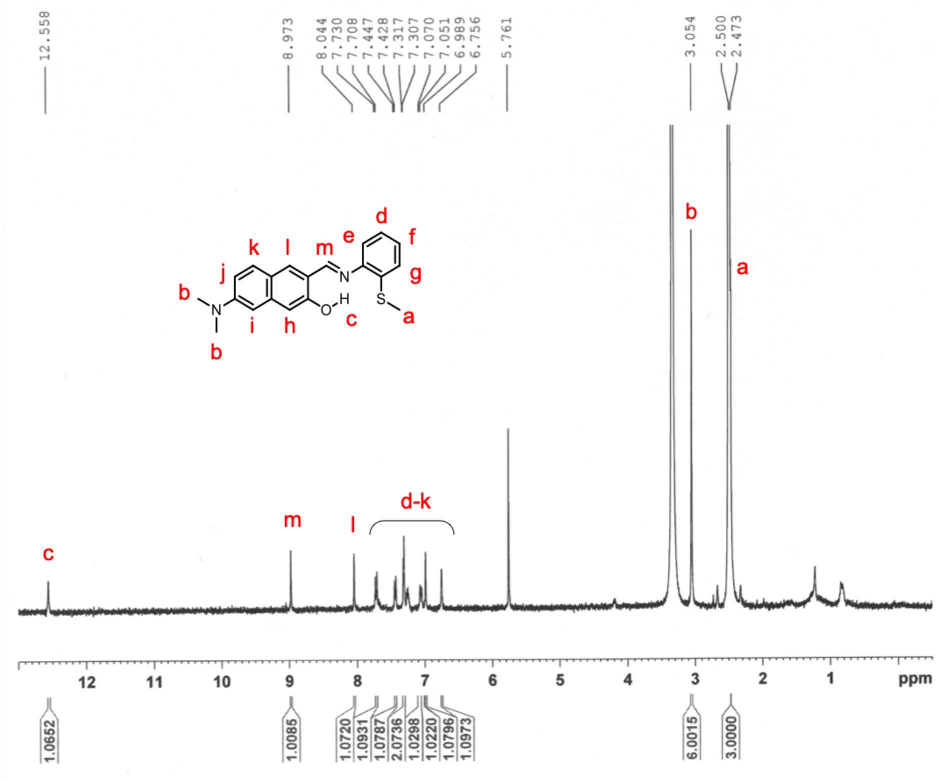

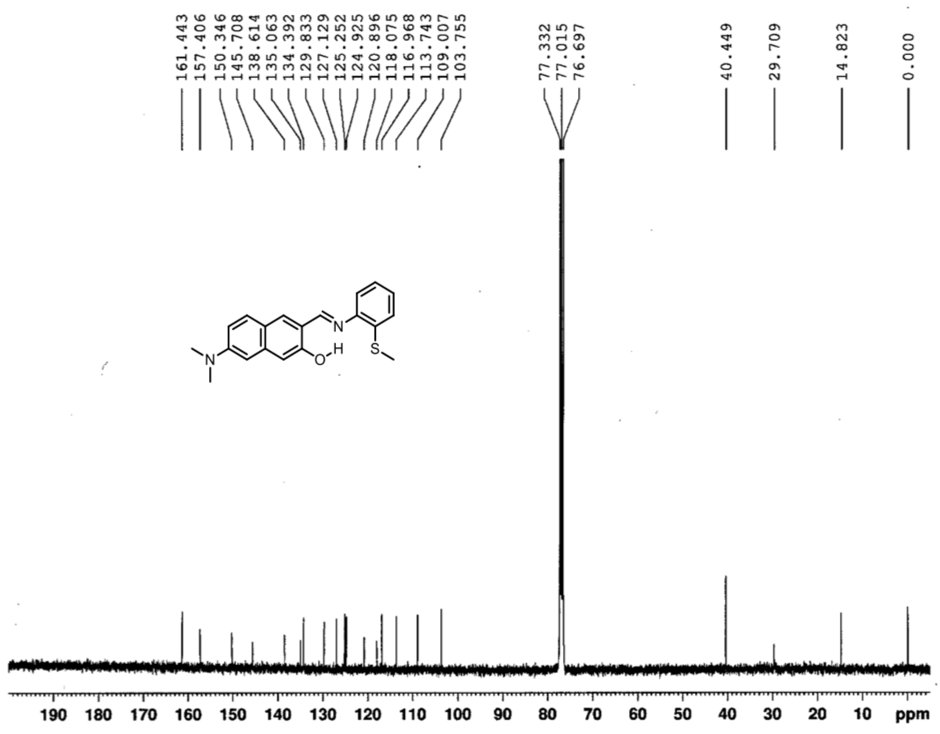

High-resolution mass spectra for FeP-1

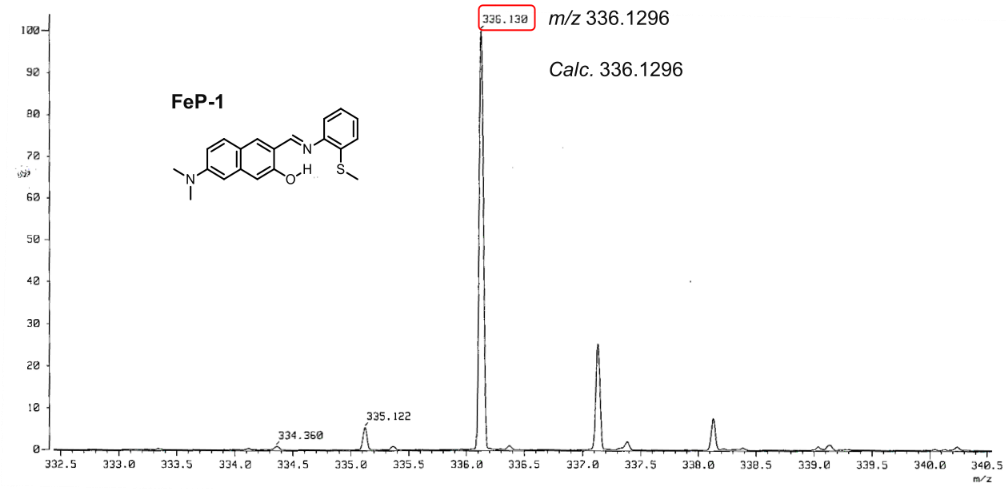

FeP-1
m/z 336.1296
Calc. 336.1296
336.130
335.122
334.360
m/z

References and Notes

Figure S1(a)

[1] C.-H. Chen, D.-J. Liao, C.-F. Wan, A.-T. Wu, Analyst, 138 (2013) 2527.

[2] S. Sinha, R.R. Koner, S. Kumar, J. Mathew, M. P. V, I. Kazi, S. Ghosh, RSC Advances, 3 (2013) 345.

[3] S. Liu, L. Zhang, W. Zan, X. Yao, Y. Yang, X. Liu, Sensors and Actuators B: Chemical, 192 (2014) 386.

[4] V.K. Gupta, A.K. Singh, L.K. Kumawat, Sensors and Actuators B: Chemical, 195 (2014) 98.

[5] Y.K. Jang, U.C. Nam, H.L. Kwon, I.H. Hwang, C. Kim, Dyes and Pigments, 99 (2013) 6.

[6] S.K. Shoora, A.K. Jain, V.K. Gupta, Sensors and Actuators B: Chemical, 216 (2015) 86.

[7] J. Zhu, Y. Zhang, L. Wang, T. Sun, M. Wang, Y. Wang, D. Ma, Q. Yang, Y. Tang, Tetrahedron Letters, 57 (2016) 3535.

Figure S1(b)

[8] H.-Y. Lin, P.-Y. Cheng, C.-F. Wan, A.-T. Wu, Analyst, 137 (2012) 4415.

[9] T.-B. Wei, P. Zhang, B.-B. Shi, P. Chen, Q. Lin, J. Liu, Y.-M. Zhang, Dyes and Pigments, 97 (2013) 297.

Figure S1(c)

[10] M. Sadia, R. Naz, J. Khan, R. Khan, Journal of Fluorescence, 28 (2018) 1281.

[11] S. Sangeeta, K. Ahmad, N. Noorussabah, S. Bharti, M.K. Mishra, S.R. Sharma, M. Choudhary, Journal of Molecular Structure, 1156 (2018) 1.

Figure S1(d)

[12] M. Shellaiah, Y.-H. Wu, A. Singh, M.V. Ramakrishnam Raju, H.-C. Lin, Journal of Materials Chemistry A, 1 (2013) 1310.

[13] B.D. Vanjare, P.G. Mahajan, S.-K. Hong, K.H. Lee, Bulletin of the Korean Chemical Society, 39 (2018) 631.

[14] G.-y. Gao, W.-j. Qu, B.-b. Shi, Q. Lin, H. Yao, Y.-m. zhang, J. Chang, Y. Cai, T.-b. wei, Sensors and Actuators B: Chemical, 213 (2015) 501.

Figure S1(e)

[15] M. Ozdemir, Y. Zhang, M. Guo, Inorganic Chemistry Communications, 90 (2018) 73.

[16] L. Long, L. Zhou, L. Wang, S. Meng, A. Gong, C. Zhang, Analytica Chimica Acta, 812 (2014) 145.

[17] H.-T. Tsai, Y.R. Bhorge, A.J. Pape, S.N. Janaki, Y.-P. Yen, Journal of the Chinese Chemical Society, 62 (2015) 316.
